# Food acquisition and consumption by NOVA food classification and lived poverty index among rural and urban households in South Africa and Ghana

**DOI:** 10.1017/S1368980024002118

**Published:** 2024-10-22

**Authors:** Nana Ama Frimpomaa Agyapong, Reginald Adjetey Annan, Florian Kroll, Charles Apprey, Linda Nana Esi Aduku, Robert Aidoo, Elizabeth Catherina Swart

**Affiliations:** 1 Department of Clinical Nutrition and Dietetics, College of Health and Allied Sciences, University of Cape Coast, Cape Coast 2331, Ghana; 2 Africa Research Universities Alliance Centre of Excellence for Non-Communicable Diseases, University of Nairobi, Nairobi 00100, Kenya; 3 Department of Biochemistry and Biotechnology, Kwame Nkrumah University of Science and Technology, Kumasi, Ghana; 4 School of Public health, University of the Western Cape, Bellville 7535, South Africa; 5 Department of Agricultural Economics, Agribusiness and Extension, Kwame Nkrumah University of Science and Technology, Kumasi 00233, Ghana; 6 Department of Dietetics and Nutrition, University of the Western Cape, Bellville 7535, South Africa

**Keywords:** NOVA food category, Poverty, Nutrition transition, South Africa, Ghana

## Abstract

**Objective:**

This study aimed to determine differences in food consumption by the NOVA food categories in South Africa and Ghana and how they relate to poverty and food supply systems.

**Design:**

This study used a cross-sectional design to assess household food acquisition and lived poverty index.

**Setting:**

The study was conducted in Khayelitsha and Mount Frere, urban and rural communities in South Africa, respectively, and Ahodwo and Ejuratia, urban and rural communities in Ghana, respectively.

**Participant:**

An adult in charge of or knowledgeable about household food acquisition and consumption was selected to participate in the study.

**Results:**

A total of 1299 households participated in the study. Supermarkets were a prominent source of ultra-processed foods for households in South Africa, while informal outlets were an important source of ultra-processed foods in Ghana. Consumption of unprocessed foods was higher among South African households (58·2 %) than Ghanaian households (41·8 %). In South Africa, deprivation was associated with increased odds of infrequent consumption of both unprocessed foods (OR 3·431 *P* < 0·001) and ultra-processed foods (OR 2·656 *P* < 0·001) compared with non-deprivation. In Ghana, no significant differences were observed between deprived households and non-deprived households in relation to the consumption of the NOVA food classes.

**Conclusion:**

Different food supply systems and poverty are associated with household acquisition of the different NOVA food classes. Policies should be geared towards formal shops in South Africa and informal shops in Ghana to reduce the consumption of key obesogenic foods.

The world is experiencing an unprecedented nutrition transition characterised by increased dependence on cheap ultra-processed foods to meet food needs. In some jurisdictions, ultra-processed foods comprise as much as 50 % of calories consumed and are associated with weight increment^(1–4)^ and chronic non-communicable diseases. In many low- and middle-income countries, it manifests as a double disease burden, implying the coexistence of infectious and non-communicable diseases^(4)^. When the problem of micronutrient deficiency is accounted for as part of the nutrition issues facing Africa, many countries now face a triple burden of disease where infectious diseases, nutritional deficiencies, obesity and related non-communicable diseases coexist. At the same time, all countries around the world currently have greater than 20 % of their adult population either overweight or obese. In some countries, the figure goes beyond 50 %^(5)^.

These epidemiological patterns correlate significantly with current food processing and supply systems dominated by ultra-processed foods, most of which contain high levels of sugar, salt, saturated fats and refined carbohydrates, a nutrient profile that promotes micronutrient deficiency and obesity^(6,7)^. Food supply systems are increasingly producing and aggressively advertising and marketing ultra-processed foods, with their highest sales increase recorded in low- and middle-income countries^(7)^. Additionally, street food consumption is common in low- and middle-income countries, especially for poorer households, and contributes to the high intake of saturated fats, sodium and sugar^(6)^. Fresh and whole foods have become an option for the rich, and in KwaZulu-Natal, for instance, the cost was reported as a major barrier to consuming fruits and vegetables^(8)^. Open markets and informal shops are a prominent source of fresh produce in many African cities^(9)^, but limited storage facilities lead to spoilage and high cost of fresh produce.

The nutrition transition disproportionately affects the poor due to the reduced availability of arable land for farming and dietary changes linked to urbanisation^(10)^. Yet, the poor are important in this transition, considering that income and food prices are the major determinants of food sourcing and other factors like proximity, transportation and the built environment^(11,12)^. The high consumption of ultra-processed foods among poor households results in the high occurrence of non-communicable diseases among them, which exacerbates their poverty^(13)^. In the sub-Saharan Africa region, urgent actions are needed to improve the dietary intake of the poor, as 42 % of the population lives in poverty. Improving their dietary patterns will reduce the burden of all-cause mortality, particularly for non-communicable diseases^(14)^.

The NOVA food classification system categorises food into four groups based on their extent of processing. The four classes include ultra-processed foods, processed foods, culinary ingredients and unprocessed foods. Unprocessed and minimally processed foods have undergone minimal alterations with no added fats, sugar and salt. Culinary ingredients are foods extracted from whole foods through processes such as refining. They include oils, fats, salt and sugar. Processed foods are foods produced by adding salt, sugar, oil or other substances to whole foods to preserve and improve their taste. Ultra-processed foods are produced through industrial formulations and usually contain one or no food ingredients but contain substances such as sugar, fats and modified starch extracted from whole foods or produced synthetically. Foods that fall under each of the classes are described in the appendix. The NOVA classification has been necessitated by the fact that the food processing level strongly correlates with disease beyond the nutrients contained in the foods^(15)^. Though concerns have been raised about the accuracy of the classification system, the NOVA has been applied successfully to population eating patterns to determine the proportion of the four food classes within the diet and how the classes are related to population disease outcomes^(15,16)^.

The emergence of supermarkets, especially in most African countries, has been shown to correlate with increased consumption of processed and ultra-processed foods. Still, some studies have shown that many African consumers source processed and ultra-processed foods from traditional markets^(6,17)^. Additionally, most food supply systems are becoming obesogenic. Considering that South Africa is one of the African countries with the most supermarkets and diminished traditional markets while Ghana is comparatively at the early stages of supermarket expansion, we aimed to determine the level of consumption of different NOVA food classes and how these are associated with different food supply systems and household lived poverty indices. Such knowledge can influence further research using longitudinal data to formulate and implement policies to improve food acquisition and promote healthy food consumption.

## Materials and methods

We used a cross-sectional approach to collect data on household socio-demographic characteristics, household food consumption and outlets for sourcing particular food items. Households were stratified based on their experience of deprivation as reflected by the lived poverty index (LPI)^(18)^. The household hunger scale was used to assess the level of hunger at the household level^(19)^.

### Study site

The study was conducted in South Africa and Ghana. In each country, one urban community and one rural community were surveyed, resulting in four study sites. The urban communities chosen were Ahodwo for Ghana and Khayelitsha for South Africa. Ahodwo is a central suburb of Kumasi, the second-largest city in Ghana. It has a population of about two million people. Ahodwo was specifically chosen because it is one of the areas with large supermarkets. Though the area is classified as a high-class residential area, it is also a residence for many urban poor. The main economic activity of the area is trading. Khayelitsha, on the other hand, is a peri-urban settlement that sprung up within the apartheid era to serve as a residence for the African population from the Western Cape – a high proportion of the population work in retail and wholesale shops or private households. Like other parts of South Africa, the area has several supermarkets, such as Shoprite, despite being home to many urban poor. The characteristics of these two urban areas made them ideal settlements for this study, especially since they have variables of interest to interrogate. Further details of the urban sites used for the study have been described elsewhere^(20)^.

For the rural communities, Ejuratia was chosen for Ghana, and Mount Frere was chosen for South Africa. Mount Frere is a rural Eastern Cape province settlement between Kokstad and Mthatha along the N2 road. It has an estimated population size of five thousand two hundred (5200) predominantly black residents. The area has poor infrastructural facilities. Ejuratia is located in the Afigya Kwabre South District of the Ashanti Region and has a population size of six thousand three hundred and twenty-one (6321) as of 2010. Compared with other parts of Ghana, the area has fewer infrastructural facilities. Farming is a major economic activity in both Ejuratia and Mount Frere. There are several supermarkets in Mount Frere, but their density is lower than that of urban areas of South Africa. In Ejuratia, there are typically small local shops called ‘provision shops’ that sell packaged and processed foods but often stock far fewer quantities than supermarkets.

### Sample size and sampling strategy

A total of 1299 households were used for the study; 675 were from South Africa, and 624 were from Ghana. The sample size, as determined by the Cochran formula, was 369 for each country^(21)^. However, in consultation with experts, study team members agreed on a sample size of 600 for each country to allow comparison and provide a good number for the different levels of study analysis. Data for the study were collected from October to the first week of December 2017. In both countries, a systematic random sampling strategy was used to select households. In Ahodwo, the town’s main streets were used to divide the locality into six parts. After a random start at each main street, every fifth household was selected to form part of the study. In Ejuratia, the main lorry station was used as the central point to divide the town into four parts, and after a random start at each point, every third household was selected. In Khayelitsha, two areas were used, namely, site B and Enkanini-Makhaza. Taxi ranks, railway stations and shopping malls in the two areas served as key points for selecting households. After a random start, every seventh household was selected to be part of the study. Six sample frames were identified in Mount Frere, incorporating a range of households from dense informal urban to scattered peri-urban and rural. The first sample frame was a remote rural settlement; another four were peri-urban frames and the more formal urban area just south of the high street. Due to the long distances and scattered nature of peri-urban and rural settlements, one household in five was sampled.

### Data collection

A pretested questionnaire was used to collect information on household socio-demographic characteristics, food consumption, food sources, household hunger and lived experiences of poverty. The questionnaire was interviewer-administered in the local dialect of participants by trained fieldworkers. The field workers were given a 3 day training for the study, after which they were made to pilot the questionnaire. Their feedback was used to finalise the questionnaire for actual data collection. The questionnaire was translated from English into the local languages of respondents by language experts from each country. All fieldworkers engaged within the two countries could speak the local languages fluently. Still, they were also trained on the translations done by the local language experts to ensure that the same wording was used during data collection. In each household, the member with the knowledge or in charge of household food acquisition and consumption was selected to respond to the questionnaire. The study was explained in detail to all respondents, and their questions were addressed before the commencement of data collection.

### Food Frequency Questionnaire (FFQ)

The household food frequency part of the questionnaire comprised a list of commonly consumed food items (see online supplementary material, Supplemental Table 1) in both study countries, and the respondent was to indicate how often each food item was consumed by the household. This consumption data covered only foods consumed within the household. The Prospective Urban Rural Epidemiology (PURE) study food frequency questionnaire, which was validated, was adapted for the study^(22,23)^. The questionnaire comprised commonly consumed foods in Ghana and South Africa. Eight (8) responses were provided for each food item ranging from ‘never’ to ‘more than 4 times per day’. The responses were coded from zero (0) to seven (7) – the FFQ collected household food consumption data over the past month. After data collection, the NOVA food classification system was used to categorise foods as unprocessed, processed, culinary ingredients (sugar) and ultra-processed. A list of food outlet types was provided for respondents to indicate where particular food items are usually purchased from. The food outlets included supermarkets, small shops, temporary stalls, fixed municipal stores, mobile vendors and container shops. The list of outlets provided reflects common outlets from which foods are sourced in South Africa and Ghana and is detailed in online supplementary material, Supplemental Table 6.

### Lived poverty index

The LPI was computed based on responses to five questions probing the frequency of various kinds of deprivation^(18)^. The questions asked included: Over the past year, how often, if ever, has the household or any of its members Gone without enough food to eat? Gone without enough clean water for use at home? Gone without medicines or medical treatment? Gone without cash income? Gone without enough fuel to cook food? A range of responses from never to always were presented with each question, and participants were asked to choose one that applied to them. Households that selected ‘never’ for all the questions were classified as non-deprived (LPI = 0), while households that reported any form of lived poverty were classified as deprived (LPI > 0).

### Household hunger scale

The household hunger scale was used to assess food insecurity within households. The questionnaire assessed how often, if ever, the household or any household member had gone without food to eat at night or during the day^(19)^. It also determined how often, if ever, there was no food in the household due to a lack of resources. The responses to these were used to classify households as experiencing no, little, moderate/severe hunger.

### Data analysis

The Statistical Package for the Social Sciences (SPSS) version 23 was used to analyse data. Descriptive statistics were used to analyse lived poverty characteristics, food consumption by the NOVA class and food sources. To determine the food outlet sources of each NOVA class, the number of households that chose a particular food outlet for foods within the specific NOVA category was computed together. This was then divided by the number of foods under the category to provide the average number of households sourcing from a particular food outlet for the specific NOVA category. *χ* ^2^ and Fisher’s exact tests were used to determine the differences in categorical variables. Multiple logistic regression was used to model the association between LPI and frequency of consumption of the NOVA food classes. A *P*-value of < 0·005 was set as statistically significant.

## Results

### Socio-demographic and household characteristics of study participants

Table 1 shows the socio-demographic and household characteristics of study respondents. The average household size was significantly higher in rural sites compared with the urban sites (Khayelitsha 3·5 (sd 2·0) *v.* Mount Frere 4·2 (sd 2·5) *P* < 0·0001; Ahodwo 3·6 (sd 2·2) *v.* Ejuratia 4·6 (sd 3·0) *P* < 0·0001). In both countries, poverty was higher in the rural areas than in urban areas.

**Table 1.** Socio-demographic characteristics of study participants

	South Africa (*n* 675)						Ghana (*n* 624)					
	Khayelitsha (*n* 326)		Mount Frere (*n* 349)				Ahodwo (*n* 320)		Ejuratia (*n* 304)			
Descriptive variables	*n*	%	*n*	%	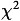	*P*-value	*n*	%	*n*	%	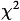	*P*-value
Gender *n* (%)												
Male	91	29·4	65	19·7	8·2	**0·004** ^*^	63	20·5	51	16·8	1·4	0·255
Female	218	70·6	265	80·3	–		245	79·5	252	83·2		
	Mean	sd	Mean	sd			Mean	sd	Mean	sd		
Age	39·3	13·0	41·0	24·5	–	0·272	42·4	15·6	42·8	16	–	0·749
Average household size	3·5	2·0	4·2	2·5		**< 0·0001** ^*^	3·6	2·2	4·6	3·0	–	**< 0·0001** ^*^
	*n*	%	*n*	%			*n*	%	*n*	%		
Non-deprived	152	46·6	131	37·6	5·6	**0·018** ^*^	227	74·4	185	61·3	12·1	**0·001** ^*^
Deprived	174	53·4	217	62·4			78	25·6	117	38·7		

* Significant at *P*-value < 0·05. Values may not add up to the total because of missing responses.

Significant *P*-values are in bold.

### Lived poverty index within households

**Figure 1.**
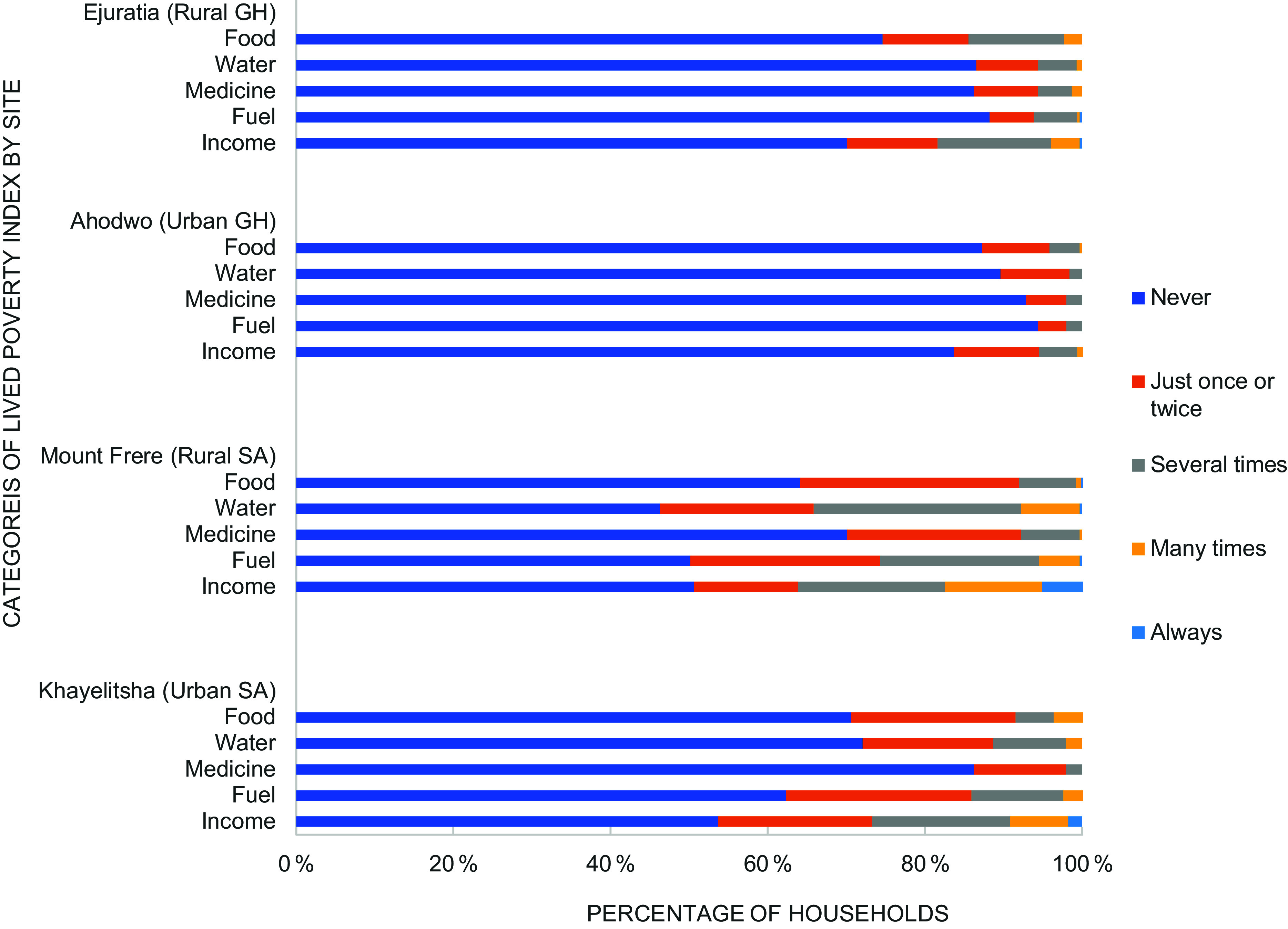
Categories of lived poverty index by site.

Figure 1 shows the percentage of households that experienced various forms of economic deprivation per lived poverty component. Overall, the most common form of poverty across all sites was the lack of income. The levels of deprivation for water and fuel were 26 % and 20 %, respectively, which occurred several times within the reporting households.

### Household hunger by lived poverty index

Table 2 shows the various levels of household hunger by lived poverty index. Hunger was significantly higher among deprived households across all sites. The form of hunger experienced by the majority of households was moderate/severe hunger.

**Table 2.** Household hunger by lived poverty index

	South Africa (*n* 675)								Ghana (*n* 624)							
	Khayelitsha *n* 326				Mount Frere *n* 349				Ahodwo *n* 320				Ejuratia *n* 304			
	Deprived		Non-deprived		Deprived		Non-deprived		Deprived		Non-deprived		Deprived		Non-deprived	
Household hunger scale	*n*	%	*n*	%	*n*	%	*n*	%	*n*	%	*n*	%	*n*	%	*n*	%
No hunger	105	60·3*	148	97·4*	151	69·6*	131	100*	47	60·3*	221	97·4*	51	43·6*	181	97·8*
Little hunger	12	6·9	2	1·3	10	4·6	0	0	12	15·4	3	1·3	20	17·1	2	1·1
Moderate/severe hunger	57	32·8	2	1·3	56	25·8	0	0	19	24·4	3	1·3	46	39·3	2	1·1
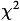	75·5				64·4				64·5				125·8			
*P*-value	**< 0·0001**				**< 0·0001**				**< 0·0001**				**< 0·0001**			

Households with no form of deprivation are referred to as non-deprived, while households that reported any form of lived poverty are referred to as deprived. Values may not add up to the total because of missing responses. *Denotes proportion which is significantly different from the others.

Significant *P*-values are in bold.

### Consumption of NOVA food categories by country

Table 3 shows the consumption of different NOVA categories by country. A higher proportion of households in South Africa (80·9 %) consumed ultra-processed foods weekly (1–6 times) compared with Ghana (19·1 %). Consumption of unprocessed foods weekly (1–6 times) was higher among South African households (58·2 %) compared with Ghanaian households (41·8 %). The consumption frequency of all the foods within each NOVA category, ranked by frequency of consumption, is presented in online supplementary material, Supplemental Tables 2–5.

**Table 3.** Consumption of the different NOVA categories by country

	South Africa (*n* 675)		Ghana (*n* 624)		South Africa (*n* 675)		Ghana (*n* 624)		South Africa (*n* 675)		Ghana (*n* 624)		South Africa (*n* 675)		Ghana (*n* 624)	
	Unprocessed foods				Processed foods				Ultra-processed foods				Sugar (culinary ingredient)			
Consumption frequency	*n*	%	*n*	%	*n*	%	*n*	%	*n*	%	*n*	%	*n*	%	*n*	%
Never/Rarely	7	28·0	18	72·0	428	54·9	352	45·1	162	26·6	448	73·4	23	11·9	171	88·1
1–3 times per month	274	47·2	307	52·8	235	51·9	218	48·1	398	76·1	125	23·9	7	9·9	64	90·1
Weekly (1–6times)	388	58·2	279	41·8	8	17·4	38	82·6	110	80·9	26	19·1	530	66·5	267	33·5
Daily (1–4 times)	0	0	0	0	0	0	0	0	0	0	0	0	111	50·7	108	49·3
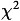	21·2				24·5				325·2				243·1			
*P*-value	< 0·0001*				< 0·0001*				< 0·0001*				< 0·0001*			

*Significant P-value < 0.05. Values may not add up to the total because of missing responses.

Significant *P*-values are in bold.

### Household consumption of different NOVA food categories by LPI

Table 4 shows household consumption of different NOVA food classes by LPI. Significant differences were observed for Khayelitsha and Mount Frere. In Khayelitsha, 79·5 % of non-deprived households consumed unprocessed foods weekly compared with 54·1 % of deprived households (*P* < 0·001). In Mount Frere, 73·1 % of non-deprived households consumed unprocessed foods weekly compared with 37·0 % of deprived households (*P* < 0·001). For processed foods, no differences were observed across all study sites. About 27 % of non-deprived households in Khayelitsha consumed ultra-processed foods weekly compared with 12·3 % of deprived households. The trend was similar for Mount Frere. For culinary ingredients (sugar), significant differences were observed only for Khayelitsha, where 61·6 % of non-deprived households consumed it weekly compared with 77·3 % of deprived households (*P* = 0·001). In Ghana, no significant differences were observed in consumption between deprived and non-deprived households.

**Table 4.** Household consumption of different NOVA class foods by lived poverty index

	South Africa (*n* 675)								Ghana (*n* 624)							
	Khayelitsha (*n* 326)				Mount Frere (*n* 349)				Ahodwo (*n* 320)				Ejuratia (*n* 304)			
	Deprived		Non-deprived		Deprived		Non-deprived		Deprived		Non-deprived		Deprived		Non-deprived	
Unprocessed foods	*n*	%	*n*	%	*n*	%	*n*	%	*n*	%	*n*	%	*n*	%	*n*	%
Never/rarely	3	1·7	1	0·7	3	1·4	0	0	1	1·3	6	2·7	5	4·3	6	3·3
1–3 times per month	76	44·2	30	19·9	133	61·6	35	26·9	36	46·2	95	43·0	68	58·1	106	57·6
Weekly (1–6 times)	93	54·1	120	79·5	80	37·0	95	73·1	41	52·6	120	54·3	44	37·6	72	39·1
Daily (1–4 times)	0	0	0	0	0	0	0	0	0	0	0	0	0	0	0	0
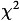	23·4				43				0·5				0·3			
*P*-value	**< 0·0001** ^*^				**< 0·0001** ^*^				0·808				0·887			
Processed foods																
Never/rarely	85	49·4	70	46·4	175	80·6	98	74·8	40	51·3	111	49·6	73	62·4	127	68·6
1–3 times per month	81	47·1	79	52·3	42	19·4	33	25·2	32	41·0	85	37·9	42	35·9	56	30·3
Weekly (1–6 times)	6	3·5	2	1·3	0	0	0	0	6	7·7	28	12·5	2	1·7	2	1·1
Daily (1–4 times)	0	0	0	0	0	0	0	0	0	0	0	0	0	0	0	0
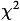	2				1·6				1·36				1·5			
*P*-value	0·381				0·226				0·496				0·500			
Ultra-processed foods																
Never/rarely	33	19·3	12	7·9	94	43·3	23	17·6	58	75·3	149	68·0	96	83·5	144	78·3
1–3 times per month	117	68·4	98	64·9	114	52·5	69	52·7	15	19·5	56	25·6	16	13·9	35	19·0
Weekly (1–6 times)	21	12·3	41	27·2	9	4·1	39	29·8	4	5·2	14	6·4	3	2·6	5	2·7
Daily (1–4 times)	0	0	0	0	0	0	0	0	0	0	0	0	0	0	0	0
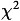	16·9				55·3				1·3				1·3			
*P*-value	**< 0·0001** ^*^				**< 0·0001** ^*^				0·517				0·498			
Culinary ingredient (sugar)																
Never/rarely	7	4·1	5	3·3	10	4·6	1	0·8	11	14·1	69	30·5	36	30·8	54	29·2
1–3 times per month	5	2·9	2	1·3	0	0	0	0	9	11·5	23	10·2	13	11·1	19	10·3
Weekly (1–6 times)	133	77·3	93	61·6	190	87·6	114	87·0	38	48·7	84	37·2	49	41·9	93	50·3
Daily (1–4 times)	27	15·7	51	33·8	17	7·8	16	12·2	20	25·6	50	22·1	19	16·2	19	10·3
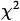	14·7				5·5				8·8				3·2			
*P*-value	**0·001** ^*^				0·064				0·0300				0·358			

* Significant at *P*-value < 0·05. Households with no form of deprivation are referred to as non-deprived, while households that reported any form of lived poverty are referred to as deprived. Values may not add up to the total because of missing responses.

Significant *P*-values are in bold.

### Household food supply source by lived poverty index

**Figure 2.**
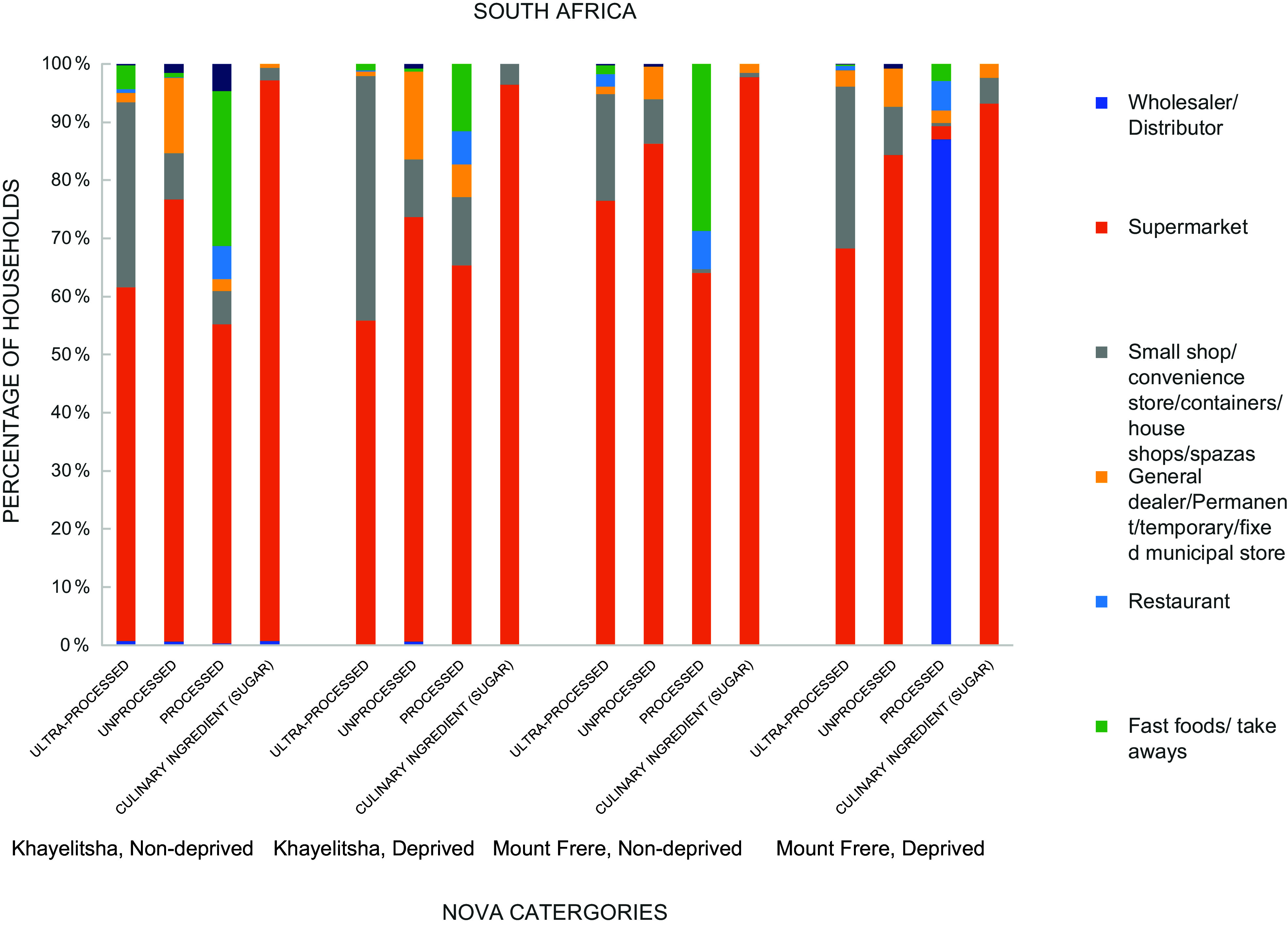
Household food supply source by lived poverty index, South Africa.

**Figure 3.**
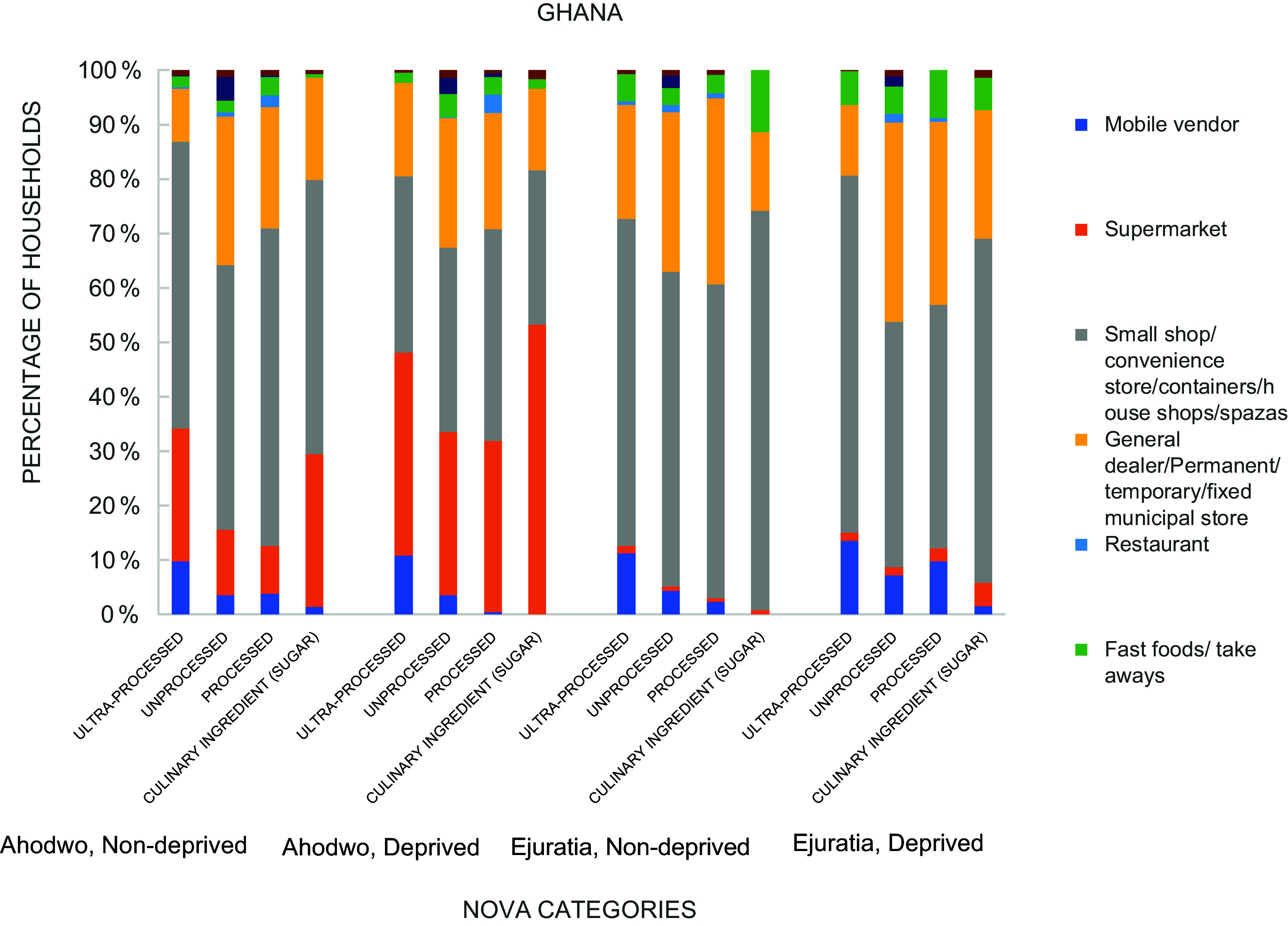
Household food supply source by lived poverty index, Ghana.

Figures 2 and 3 show the food supply source of the NOVA food categories by lived poverty index. Supermarket was the most frequently sourced food outlet for households in South Africa for all categories of the NOVA (Fig. 2). In South Africa, supermarkets were the most prominent source of food. About 60·5 % of non-deprived households in Khayelitsha sourced ultra-processed foods from supermarkets. Among deprived households, the proportion was 55·7 %. The trend was similar for Mount Frere. In Ghana, informal sources such as small shops, temporary stalls, container shops and permanent stalls are important food sources. Most non-deprived households in Ahodwo (52 %) sourced ultra-processed foods frequently from small shops/convenience stores. Among deprived households, supermarkets were a frequent source (36·9 %). In Ejuratia, non-deprived (56·1 %) and deprived (65·8 %) households frequently sourced ultra-processed foods from small shops/convenience stores.

### Relationship between LPI and NOVA food category consumption

Tables 5 and 6 show the multiple logistic regression of LPI and NOVA food category consumption frequency. In South Africa, deprived households had significantly increased odds of consuming unprocessed foods (OR 3·431, *P* < 0·001) and ultra-processed foods infrequently (OR 2·656, *P* < 0·001) compared with non-deprived households. The associations observed between the lived poverty index and the consumption of the NOVA food categories were not statistically significant for households in Ghana.

**Table 5.** Relationship between lived poverty index and NOVA food category consumption (South Africa)

South Africa	Explanatory variables	OR	
Lived poverty	Unprocessed foods		
Not Deprived = 0^†^	Frequent = 0^†^		
Deprived = 1	Infrequent = 1	3·431	2·293–5·133***
	Processed foods		
	Frequent = 0^†^		
	Infrequent = 1	0·198	0·030–1·302
	Ultra-processed foods		
	Frequent = 0^†^		
	Infrequent = 1	2·656	1·528–4·618***
	Culinary ingredient (sugar)		
	Frequent = 0^†^		
	Infrequent = 1	1·230	0·456–3·318
	Number of household members	0·968	0·886–1·057
	Age of respondent	0·974	0·961–0·987***
	Gender of respondent		
	Male = 0^†^		
	Female = 1	1·128	0·715–1·779
	Household hunger		
	Moderate/severe hunger = 0^†^		
	Little hunger	0·138	0·018–1·080
	No hunger	0·017	0·004–0·072***

***P ≤ 0·001. Cox and Snell R-squared = 0·289. Nagelkerke R-squared = 0·391. Households with no form of deprivation are referred to as non-deprived, while households that reported any form of lived poverty are referred to as deprived.

^†^Variable set as reference.

**Table 6.** Relationship between lived poverty index and NOVA food category consumption (Ghana)

Ghana	Explanatory variables	OR	
Lived poverty	Unprocessed foods		
Not deprived = 0^†^	Frequent = 0^†^		
deprived = 1	Infrequent = 1	1·136	0·703–1·835
	Processed foods		
	Frequent = 0^†^		
	Infrequent = 1	1·341	0·476–3·776
	Ultra-processed foods		
	Frequent = 0^†^		
	Infrequent = 1	1·447	0·436–4·801
	Culinary ingredient (sugar)		
	Frequent = 0^†^		
	Infrequent = 1	1·026	0·635–1·660
	Number of household members	1·113	1·023–1·212**
	Age of respondent	0·992	0·978–1·007
	Gender of respondent		
	Male = 0^†^		
	Female = 1	1·845	0·960–3·548
	Household hunger		
	Moderate/severe hunger = 0^†^		
	Little hunger	0·521	0·069–3·951
	No hunger	0·008	0·002–0·032***

***P ≤ 0·001, **P < 0·05. Cox and Snell R-squared = 0·336. Nagelkerke R-squared = 0·468. Households with no form of deprivation are referred to as non-deprived, while households that reported any form of lived poverty are referred to as deprived.

^†^Variable set as reference.

## Discussion

This paper assessed the lived poverty experiences of rural and urban South Africans and Ghanaians and how they relate to food acquisition at the household level and consumption of different NOVA food categories. Findings from this study indicate that many rural dwellers continue to live in deprived conditions and lack basic amenities, and deprivation is strongly associated with household hunger^(24)^. The mechanisms by which urbanisation results in unhealthy dietary patterns include increasing the reliance on cash to obtain food in poor urban areas where food safety nets like kinsmen are unavailable to provide food in periods of economic distress. Popkin^(25)^ documented that the positive trend in BMI observed across the globe is largely attributable to sharp positive increases in BMI in rural areas. The lived poverty index found in this study was moderate for both South Africa and Ghana, as also reported by Mattes *et al*.^(26)^. Policies that reduce geographic isolation through infrastructure development, subsidising basic amenities and increasing the availability of higher-skilled jobs in rural areas, can help alleviate rural poverty and deprivation^(27)^.

Overall, South African households had higher consumption of ultra-processed foods, unprocessed foods and sugar. Non-deprived households across all sites consumed unprocessed and ultra-processed foods more frequently than deprived households, but this was statistically significant only for South African households. This implies that households consume ultra-processed and unprocessed foods as living conditions improve. The findings on consumption may reflect dietary adaptations households make as they transition in and out of poverty, considering that the lived poverty construct used in this study is non-static poverty^(28)^. Consumption of ultra-processed foods, even along with unprocessed foods, can increase the risk of obesity and related non-communicable diseases. In South Africa, the consumption of tea and coffee is high, and sugar and milk are often added to make them tastier. The frequent sugar intake is further worsened by consuming sugar-sweetened beverages. This pattern of intake is of grave health concern, considering its impact on weight gain and the rising obesity and non-communicable disease burden in South Africa and Ghana. Research conducted by Mbogori *et al*.^(29)^ using data from Ghana, South Africa, Malawi and Kenya reported that nutritional shifts occurring in Africa, especially those related to overweight and obesity, are driven by economic developments. Furthermore, we observed that the consumption of fresh fruits and vegetables was higher among non-deprived households, while deprived households consumed cooked vegetables more frequently. Cooked vegetables such as onion, pepper, eggplants and tomatoes form the basis for preparing many gravies and soups in Ghana and South Africa. Loss of vitamins during cooking and the addition of oils in the preparation of gravies make raw vegetables more beneficial than cooked or fried vegetables. Additionally, staple vegetables that are usually cooked are cheaper than exotic vegetables and fruits, which are usually eaten raw, and this may account for their higher consumption among deprived households.

The acquisition of food from traditional outlets like small shops, fixed municipal stores, permanent stalls, container shops and mobile vendors may be associated with insignificant differences in consumption among the deprived and non-deprived in African countries, irrespective of their stage in the nutrition transition. In contrast, acquiring foods from formal outlets like supermarkets and multinational food companies results in higher differences in consumption among the deprived and non-deprived. South Africa is ahead of Ghana in the nutrition transition^(30,31)^. In Africa, the characteristics of the nutrition transition include establishing and expanding supermarkets and multinational food chains. In South Africa, this form of nutrition transition is very noticeable. It is associated with an increased consumption of both unprocessed and ultra-processed foods but more for ultra-processed foods and resultant high prevalence of overweight and obesity^(29)^. Results from this study show that deprived households in South Africa had significantly reduced odds of consuming processed and ultra-processed foods compared with non-deprived households. In Ghana, no significant association was observed. In Ghana, traditional outlets like small shops and municipal stores were an important food source among the deprived and non-deprived. This may have been reflected in the very few consumption differences observed. However, cold storage facilities exist in these traditional markets for fish, other animal products and even sugar-sweetened beverages, but not fresh fruits and vegetables. These are, therefore, sold in small quantities, and where quantities are high, a lot of wastage occurs, leading to their scarcity, exorbitant cost and low consumption^(32,33)^.

In South Africa, where supermarkets served as a prominent food source, the differences observed between the deprived and non-deprived were higher, thus supporting the assertion that supermarket expansion in Africa does not support food security for all persons^(33)^. In small towns of Kenya, research indicates that supermarket shopping is linked to higher household acquisition of ultra-processed foods than whole foods, especially among middle-class and rich households^(32,33)^. Traditional food outlets serve as a major food source for the African population, which is food insecure, and their replacements with supermarkets may plunge poor households into food insecurity along with limited consumption of whole and unprocessed foods^(34)^. On the other hand, formal food systems allure the rich to have a high frequency of consumption of obesogenic food. Therefore, the African population is at risk of malnutrition irrespective of their living conditions, and nutrition policies should target the poor and rich alike and not only focus on the poor. African countries will likely benefit from strategic policies strengthening the informal retail system typical of African food supply environments.

Local government authorities can incentivize more frequent purchases and consumption of healthy food by strengthening and protecting the informal systems by easing public trading regulations for healthy food traders. Informal fruit and vegetable value chains could be supported by providing subsidies for cold chain facilities, training on storage or minimal processing facilities to producers and traders. These interventions have the potential to make healthy foods affordable to both the rich and the poor while reducing the acquisition and consumption of unhealthy foods. These actions are more practical in Ghana, considering that its food supply system is not as dominated by supermarkets as in South Africa. For ultra-processed foods, policies should mandate upstream regulation by taxing raw ingredients and limiting amounts of ingredients such as sugar and fat. Without these regulations, the informal food supply system will continue to adapt its sales in response to the demands for cheap ultra-processed food and further reduce the sale and consumption of healthy staples.

### Strengths and limitations of the study

The main strength of this study is that it presents findings on how the lived poverty index and food supply systems are associated with household acquisition of different NOVA foods in two different country contexts. The findings are essential for the formulation of policies that target ultra-processed consumption. The FFQ collected food consumption data at the household level. Foods consumed by individual household members outside the home were not assessed in this study. This is a limitation as consumption data may not reflect typical food consumption by household members. How households process foods such as eggs, meat, chicken and nuts before consumption was also not assessed, and this limits the categorisation of these food items under the NOVA. Processed meat stock and bouillon cubes may also be used in the preparation of meat and other animal flesh; this limits their categorisation under unprocessed foods.

### Conclusion

The findings of this paper show that deprived households across all study sites experienced higher rates of household food insecurity. In South Africa, where supermarkets were a prominent food source, consumption of ultra-processed foods was higher than in Ghana, where informal outlets were key food sources. The results further show how households lived poverty and food supply system is related to food acquisition at the household level. In South Africa, deprived households had a lower frequency of consumption of unprocessed and ultra-processed foods than non-deprived households. In Ghana, differences observed between deprived and non-deprived households were marginal and insignificant. Findings from this study show that household-level deprivation or non-deprivation affects food acquisition and consumption. Still, differences observed among households regarding these consumption patterns are influenced by existing main food supply systems, with the formal food system (supermarkets) magnifying these differences. Therefore, more research and longitudinal studies are needed to establish these associations and linkages to drive the implementation of policies to improve healthy food consumption.

## Supporting information

Agyapong et al. supplementary material Agyapong et al. supplementary material

## Supplementary material

For supplementary material accompanying this paper, visit https://doi.org/10.1017/S1368980024002118
