# Supplementary material for: Food acquisition and consumption by NOVA food classification and lived poverty index among rural and urban households in South Africa and Ghana
Source: Public Health Nutr. 2024 Oct 22;27(1):e260. doi: 10.1017/S1368980024002118 (PMC11705029; doi:10.1017/S1368980024002118)
Supplement: Agyapong et al. supplementary material [file S1368980024002118sup001.docx]

**APPENDIX**

**Supplementary Table 1. Foods under each of the NOVA categories included in the FFQ.**

| UNPROCESSED FOODS CATERGORY | |
| --- | --- |
| **Foods** | **Description** |
| Milk, amasi, curdled milk | Includes whole milk and products from whole milk such as full cream, low fat and fat free milk, yoghurt (unsweetened), amasi, or curdled milk |
| Organ meat | All types of organ meat |
| Fish | Includes any type of fish that is steamed, boiled, grilled, barbecued, roasted or baked |
| Fruit | All types of fresh and raw fruits |
| Raw vegetables | Fresh and raw, excludes potato |
| Cooked vegetables | Cooked with water, no oil added |
| Legumes | Includes dried beans, peas, lentils, soybeans |
| Rice | Includes sticky rice, rice noodle, white rice, brown rice |
| Root tubers | Potatoes, yam, sweet potato, cassava, plantain (boiled or roasted; cooked without oil) |
| Maize | Includes maize meal, samp and cooked mealies |
| Nuts | Peanuts & tree nuts |
| Tea/coffee | All kinds of teabags and coffee |
| Chicken | All types of chicken |
| Meat | Includes beef, pork, lamb, mutton, goat, veal consumed as main dish or as sandwich or mixed dish |
| Eggs | Boiled or fried |
| PROCESSED FOODS CATEGORY | |
| **Foods** | **Description** |
| Fried fish | All types of fish fried in oil |
| Salted fish | Salted fish (dried and fermented) |
| Pot fried | Deep fried potatoes, hot chips, fried yam, fried cocoyam |
| Pasta | All types of pasta |
| Homemade bread | includes chapati, paratha, roti, gluten bread, steamed bread, bread rolls, (in Ghana the range of sweet bread, grain bread etc) |
| Fried vegetables | Fried or stir fried in oil |
| ULTRA-PROCESSED FOODS CATEGORY | |
| **Foods** | **Description** |
| Processed milk | Flavoured & sweetened milk/youghut, condensed/ideal milk, powdered milk, non-dairy creamers and other alternative types of milk |
| Processed meat | Includes polony, sausage |
| Instant noodles | All types of instant noodles |
| Commercial bread | Chapati, paratha, roti, gluten bread, steamed bread, bread rolls, (in Ghana the range of sweet bread, grain bread etc) |
| Vetoek | Flour based though with sugar and salt that is deep fried in oil. |
| Margarine | Fat spreads and all types including butter |
| Commercial breakfast cereals | Includes cornflakes, Weetabix, instant maize meal |
| Sweets | Includes all types of chocolate, candy, drops, sweetened bars etc. |
| Confectionery | Sugary baked goods, biscuits and related food items |
| Salty snacks | Includes chips, crisps, puffs, plantain chips and other food similar to these |
| Sugar-sweetened beverage | includes Coke, Pepsi, delister, Guarana, Gingerale, Fanta, Freezit, Thums up. Sweetened sports drinks. Concentrates (powder and liquid) that will be mixed with water |
| Diet beverage | Diet version of drinks like Coke, Pepsi, delister, Guarana, Gingerale, Fanta, Freezit (light and regular), Thums up. Sweetened sports drinks. Concentrates (powder and liquid) that will be mixed with water |
| CULINARY INGREDIENTS | |
| Sugar | All types of sugar |

**Supplementary Table 2. Consumption of unprocessed foods ranked by frequency of consumption (South Africa)**

| **Ranking of foods** | **Khayelitsha**  **Not deprived**  **n=152** | **n (%)** | **Khayelitsha**  **Deprived**  **n=174** | **n (%)** | **Mount Frere**  **Not deprived**  **n=131** | **n (%)** | **Mount Frere**  **Deprived**  **n=217** | **n (%)** |
| --- | --- | --- | --- | --- | --- | --- | --- | --- |
| 1 | Rice | 146 (96.1) | Rice | 160 (93.0) | Rice | 127 (96.9) | Rice | 203 (93.6) |
| 2 | Tea/coffee | 135 (88.8) | Tea/coffee | 137 (79.6) | Maize | 122 (93.1) | Maize | 199 (91.7) |
| 3 | Maize | 130 (85.6) | Cooked vegetables | 133 (77.4) | Root tubers | 105 (80.1) | Tea/coffee | 182 (83.9) |
| 4 | Fruit | 129 (84.9) | Chicken | 127 (73.0) | Chicken | 99 (75.6) | Root tubers | 166 (76.5) |
| 5 | Chicken | 129 (84.8) | Root tubers | 126 (72.1) | Fruit | 89 (68.0) | Chicken | 146 (67.2) |
| 6 | Root tubers | 116 (76.4) | Maize | 119 (69.2) | Milk | 84 (64.2) | Cooked vegetables | 141 (64.8) |
| 7 | Milk | 107 (70.5) | Fruit | 115 (66.9) | Cooked vegetables | 80 (61.0) | Fish | 126 (53.1) |
| 8 | Raw vegetables | 85 (55.9) | Eggs | 85 (48.9) | Eggs | 64 (48.9) | Eggs | 126 (53.1) |
| 9 | Eggs | 84 (55.3) | Milk | 80 (46.0) | Fish | 54 (41.2) | Milk | 83 (38.4) |
| 10 | Cooked vegetables | 76 (50.0) | Raw vegetables | 46 (26.8) | Raw vegetable | 40 (30.5) | Fruit | 78 (36.0) |
| 11 | Fish | 39 (25.7) | Fish | 40 (23.3) | Legumes | 38 (29.0) | Meat | 26 (12.0) |
| 12 | Legumes | 32 (21.1) | Meat | 24 (13.8) | Organ meat | 12 (9.2) | Raw vegetables | 24 (10.2) |
| 13 | Meat | 28 (18.4) | Legumes | 12 (7.0) | Meat | 11 (8.4) | Raw vegetables | 11 (5.1) |
| 14 | Organ meat | 28 (18.4) | Nuts | 12 (7.0) | Nuts | 5 (3.9) | Legumes | 9 (4.2) |
| 15 | Nut | 21 (13.8) | Organ meat | 9 (5.2) | Tea/coffee | 1 (0.8) | Nuts | 8 (3.7) |

**Supplementary Table 3. Consumption of unprocessed foods ranked by frequency of consumption (Ghana)**

| **Ranking of foods** | **Ahodwo**  **Not Deprived**  **n=227** | **n(%)** | **Ahodwo**  **Deprived**  **n= 78** | **n(%)** | **Ejuratia**  **Not deprived**  **n=185** | **n(%)** | **Ejuratia**  **Deprived**  **n=117** | **n(%)** |
| --- | --- | --- | --- | --- | --- | --- | --- | --- |
|  | Rice | 196(87.0) | Rice | 66(84.6) | Rice | 144(83.2) | Fish | 99(84.6) |
|  | Fish | 165(73.1) | Fish | 64(82.1) | Root tubers | 152(82.1) | Root tubers | 98(83.7) |
|  | Fruit | 159(70.6) | Root tubers | 48(61.6) | Maize | 145(78.3) | Maize | 91(77.8) |
|  | Root tubers | 134(59.3) | Maize | 47(60.3) | Fish | 125(67.5) | Rice | 86(73.5) |
|  | Maize | 131(57.9) | Fruit | 46(59.0) | Fruit | 123(66.5) | Fruit | 74(63.2) |
|  | Raw vegetables | 115(50.9) | Cooked vegetables | 43(55.2) | Cooked vegetables | 119(64.3) | Cooked vegetables | 68(58.1) |
|  | Eggs | 114(50.7) | Raw vegetables | 39(50.1) | Eggs | 83(44.9) | Eggs | 33(28.3) |
|  | Cooked vegetables | 103(45.6) | Eggs | 39(50.0) | Tea/coffee | 60(32.4) | Raw vegetables | 30(25.6) |
|  | Tea/coffee | 89(39.4) | Tea/coffee | 30(38.5) | Legumes | 57(30.8) | Nuts | 28(23.9) |
|  | Milk | 77(34.0) | Milk | 17(21.7) | Raw vegetables | 49(26.4) | Tea/coffee | 27(23.1) |
|  | Meat | 70(30.8) | Meat | 15(19.2) | Nuts | 44(24.0) | Meat | 22(18.8) |
|  | Chicken | 61(27.0) | Legumes | 13(16.7) | Chicken | 41(22.1) | Legumes | 18(15.4) |
|  | Organ meat | 29(12.7) | Chicken | 12(15.4) | Meat | 40(21.6) | Chicken | 18(15.4) |
|  | Legumes | 52(23.0) | Nuts | 10(12.8) | Milk | 18(9.7) | Organ meat | 6(5.1) |
|  | Nuts | 23(10.2) | Organ meat | 8(10.3) | Organ meat | 13(6.0) | Milk | 5(4.3) |

**Supplementary Table 4. Consumption of processed and ultra-processed foods ranked by frequency of consumption (South Africa)**

| **Rankings of foods** | **Khayelitsha**  **Not deprived**  **n=152** | **n (%)** | **Khayelitsha**  **Deprived**  **n= 174** | **n (%)** | **Mount Frere**  **Not deprived**  **n=131** | **n(%)** | **Mount Frere**  **Deprived**  **n=217** | **n (%)** |
| --- | --- | --- | --- | --- | --- | --- | --- | --- |
| **Ultra-processed foods** | | | | | | | | |
| 1 | Margarine | 135 (89.4) | Margarine | 137 (79.6) | Margarine | 115 (87.8) | Margarine | 146 (67.2) |
| 2 | Commercial bread | 128 (84.8) | Commercial bread | 122 (70.9) | Sugar-sweetened beverage | 96 (73.3) | Commercial bread | 132 (60.8) |
| 3 | Sugar sweetened beverages | 121 (80.1) | Breakfast cereals | 87 (50.6) | Commercial bread | 77 (58.8) | Sugar-sweetened beverages | 89 (41.0) |
| 4 | Breakfast cereals | 84 (55.7) | Sugar-sweetened beverages | 74 (43.1) | Salty snack | 60 (45.9) | Breakfast cereals | 89 (41.0) |
| 5 | Processed meat | 82 (53.9) | Processed meat | 70 (40.8) | Processed meat | 59 (45.1) | Processed meat | 69 (31.8) |
| 6 | Processed milk | 47 (30.9) | Processed milk | 34 (19.6) | Breakfast cereals | 54 (41.3) | Processed milk | 36 (16.6) |
| 7 | Salty snack | 42 (27.9) | Salty snack | 33 (19.2) | Processed milk | 35 (26.8) | Salty snacks | 23 (10.6) |
| 8 | Instant noodles | 40 (26.5) | Vetoek | 28 (16.3) | Confectionery | 31 (23.6) | Vetoek | 23 (10.6) |
| 9 | Sweets | 26 (17.2) | Sweets | 23 (13.3) | Instant noodles | 25 (19.1) | sweets | 18 (8.3) |
| 10 | Vetoek | 24 (15.8) | Instant noodles | 14 (8.1) | Sweets | 25 (19.1) | Confectionery | 11 (5.1) |
| 11 | Confectionery | 23 (15.3) | Confectionery | 11 (6.4) | Vetoek | 17 (13.0) | Instant noodles | 3 (1.4) |
| 12 | Diet beverage | 6 (4.0) | Diet beverages | 3 (1.8) | Diet beverages | 1 (0.8) | Diet beverages | 2 (0.9) |
| **Processed foods** | | | | | | | | |
| 1 | Homemade bread | 36 (24) | Homemade bread | 59 (34.4) | Homemade bread | 56 (42.8) | Homemade bread | 114 (52.6) |
| 2 | Pot fried | 24 (15.8) | Pot fried | 25 (14.5) | Pot fried | 10 (7.6) | Pot fried | 19 (8.7) |
| 3 | Pasta | 20 (13.3) | Fried vegetables | 22 (12.8) | Pasta | 10 (7.6) | Fried vegetables | 9 (4.2) |
| 4 | Fried fish | 14 (9.3) | Pasta | 17 (9.9) | Fried fish | 2 (1.5) | Pasta | 4 (1.8) |
| 5 | Fried vegetables | 11 (7.3) | Fried fish | 12 (7) | Fried vegetables | 2 (1.5) | Fried fish | 2 (0.9) |
| 6 | Salted Fish | 1 (0.7) | Salted fish | 0 (0) | Salted fish | 0 (0) | Salted fish | 0 (0) |

**Supplementary Table 5. Consumption of processed and ultra-processed foods ranked by frequency of consumption (Ghana)**

| **Rankings of foods** | **Ahodwo**  **Not deprived**  **n-227** | **n (%)** | **Ahodwo**  **Deprived**  **n-78** | **n (%)** | **Ejuratia**  **Not deprived)**  **n=185** | **n (%)** | **Ejuratia (**  **Deprived**  **n=117** | **n (%)** |
| --- | --- | --- | --- | --- | --- | --- | --- | --- |
|  | Ultra-processed |  |  |  |  |  |  |  |
| 1 | Commercial bread | 150 (66.3) | Commercial bread | 37 (47.4) | Commercial bread | 91(49.2) | Commercial bread | 64(54.6) |
| 2 | Sugar-sweetened beverage | 70 (31.0) | Sugar-sweetened beverage | 20 (25.7) | Confectionery | 37(19.9) | Breakfast cereals | 26 (22.3) |
| 3 | Confectionery | 54(23.9) | Confectionery | 18(23.1) | Breakfast cereals | 34 (18.4) | Processed milk | 15 (12.9) |
| 4 | Processed milk | 38(16.7) | Instant noodles | 15 (19.2) | Processed milk | 27 (14.7) | Sugar-sweetened beverages | 13 (11.2) |
| 5 | Breakfast cereals | 37(16.4) | Margarine | 9(11.5) | Sugar sweetened beverage | 27 (14.6) | Confectionery | 13 (11.1) |
| 6 | Instant noodles | 28(12.5) | Processed milk | 8 (10.3) | Instant noodles | 16 (8.6) | Margarine | 7 (6.1) |
| 7 | Margarine | 26(11.5) | Breakfast | 7 (8.9) | Sweets | 16 (8.6) | Instant noodles | 6 (5.1) |
| 8 | Sweets | 21 (9.4) | Salty snacks | 6(7.7) | Salty snacks | 16 (18.6) | Salty snacks | 3 (2.6) |
| 9 | Salty snacks | 12 (5.4) | Sweets | 5(6.5) | Margarine | 12 (6.5) | Sweets | 2 (1.7) |
| 10 | Diet beverages | 11(4.9) | Diet beverage | 4 (5.1) | Processed meat | 5(2.7) | Vetoek | 1 (0.9) |
| 11 | Processed meat | 7 (3.1) | Vetoek | 2(2.6) | Diet beverage | 2 (1.1) | Diet beverages | 1 (0.9) |
| 12 | Vetoek | 2 (0.9) | Processed meat | 0 (0) | Vetoek | 0 (0) | Processed meat | 0 (0) |
|  | **Processed foods** |  |  |  |  |  |  |  |
| 1 | Fried fish | 95(42.5) | Fried fish | 28 (35.8) | Fried fish | 60 (32.4) | Fried fish | 40(34.2) |
| 2 | Salted fish | 63(27.8) | Homemade bread | 23 (29.5) | Salted fish | 53 (28.6) | Salted fish | 32 (27.4) |
| 3 | Homemade bread | 53 (23.4) | Salted fish | 16 (20.6) | Homemade bread | 36 (19.4) | Pasta | 15 (12.8) |
| 4 | Fried vegetables | 35 (15.6) | Fried vegetables | 11 (14.1) | Pasta | 16 (8.7) | Fried vegetables | 13 (11.1) |
| 5 | Pasta | 28(12.4) | Pot fried | 4 (5.2) | Fried vegetables | 14 (7.5) | Homemade bread | 8 (6.8) |
| 6 | Pot fried | 19 (8.4) | Pasta | 4 (5.2) | Pot fried | 6(3.2) | Pot fried | 4 (3.5) |

**Supplementary Table 6. Definition of food outlets**

| **Food outlet** | **Definition** |
| --- | --- |
| Wholesaler / Distributor | Sale of foods, food products and other products in bulk like boxes or cartons (eg. Makro) |
| Supermarket | Large retail shops that offer wide variety of local and exotic foods and other products which are grouped into sections eg. Shoprite |
| Small shop/ convenience store/containers/house shops/spazas | Dedicated shop with a sign that sell everyday household food and non-food items. Informal shop attached to or part of a home with no sign; not dedicated to food retail only. A stand-alone building with a sign; trading bulk groceries and a large variety of non-food items. The container shops usually sell convenience foods like biscuits, sugar-sweetened beverages, etc. They also sell non-food items |
| General dealer/Permanent/temporary/fixed municipal store | Fixed municipal stall refers to shops owned and regulated by the municipal/local government authority and the permanent or temporary stall refers a permanent or temporary structure that may be privately owned. The general dealer shops sell variety of food and non-food items. |
| Mobile trader | Foods sold on trolleys, wheelbarrows; carry-tray, baskets or buckets |
| Restaurant | Sit-down meals with service |
| Fast foods/ take away | Ready-to-eat, quickly-served meals are usually not eaten at the place of service. |
| Informal abattoir | Slaughter facilities that sell animal flesh |
